# Case report: Dynamic genetic profiles reveal a patient with myelodysplastic neoplasm transforming into acute myeloid leukemia

**DOI:** 10.1007/s12672-026-04863-y

**Published:** 2026-03-27

**Authors:** Guiying Guo, Huanchen Cheng, Meng Sun, Lixia Liu, Jiayue Qin, Yu Liu, Tiejun Gong

**Affiliations:** 1 Institute of Harbin Hematology and Oncology, The First Hospital of Harbin, No. 149 Diduan Street, Harbin, 150010 China; 2 Department of Medical Affairs, Acornmed Biotechnology Co., Ltd, Floor 19, Block 5, Yard 18, Kechuang 13 RD, Beijing, 100176 China

**Keywords:** MDS, AML, Mutation, VAF, Case report

## Abstract

A 62-year-old male presented with fatigue, dizziness, palpitations, and pancytopenia, subsequently diagnosed with myelodysplastic neoplasm (MDS) with increased blasts 2, alongside mutations in *DDX41* and *TP53* via a targeted 521-gene hematologic disorder panel. Treatment commenced with azacitidine and supportive care, leading to transient complete remission, and the two related mutations were eliminated. However, disease relapse occurred with the emergence of a novel *SETBP1* mutation, accompanied by two mutations detected at diagnosis. Despite adjustments in the treatment regimen, such as adding daratumumab, the patient’s disease progressed to secondary acute myeloid leukemia (sAML) with increased variant allele frequencies in three mutations. This indicated the novel *SETBP1* mutation might be associated with AML transformation. Ultimately, after discontinuing treatment, the patient passed away. This case indicates three co-mutations, including *SETBP1*, *DDX41* and *TP53*, may led to rapid disease progression, and underscores the challenges in managing MDS progressing to sAML, highlighting the prognostic value of genetic mutations detected through multiple genetic tests and the need for further treatment strategies.

**Supplementary Information:**

The online version contains supplementary material available at 10.1007/s12672-026-04863-y.

## Introduction

Myelodysplastic neoplasms (MDS) are a heterogeneous group of clonal hematopoietic disorders characterized by ineffective hematopoiesis, leading to peripheral blood cytopenias and an increased risk of progression to acute myeloid leukemia (AML) [1]. MDS is often associated with various genetic mutations, which contribute to its pathophysiology and clinical manifestations [2, 3]. The 5th edition of the World Health Organization (WHO) classifies MDS based on the percentage of blasts in the bone marrow, with increased blasts indicating a higher risk of transformation to secondary AML (sAML) [4]. The diagnosis of MDS typically involves a combination of clinical evaluation, complete blood count (CBC), bone marrow (BM) examination, and genetic testing aimed at identifying specific mutations that may influence prognosis and treatment strategies [5].

The progression from MDS to sAML is a critical concern in hematology, indicating a shift in disease biology and treatment requirements [6]. Patients with sAML often exhibit distinct clinical features, and have more monogenic adverse prognostic factors compared to those with de novo AML [7]. Mutations in genes such as *TP53* are associated with poor prognosis and treatment resistance, complicating management strategies. Current therapeutic approaches for MDS and sAML include hypomethylating agents, chemotherapy, and targeted therapies. However, the optimal treatment strategy has not yet been established and is still under active investigation [8].

In this case report, we present a 62-year-old male patient diagnosed with MDS who subsequently progressed to sAML. We reported for the first time the case with three co-mutations, including *SETBP1*, *DDX41* and *TP53*, which led to rapid disease progression. This case is particularly noteworthy due to the identification of clonal evolution via eight multi-gene panel tests, and the patient’s complex treatment journey. The rarity of such a progression highlights the importance of considering MDS as a potential precursor to sAML in patients presenting with genetic findings. Understanding the clinical implications of this case may help improve diagnostic and therapeutic strategies for similar patients in the future.

## Case presentation

**Fig. 1:**
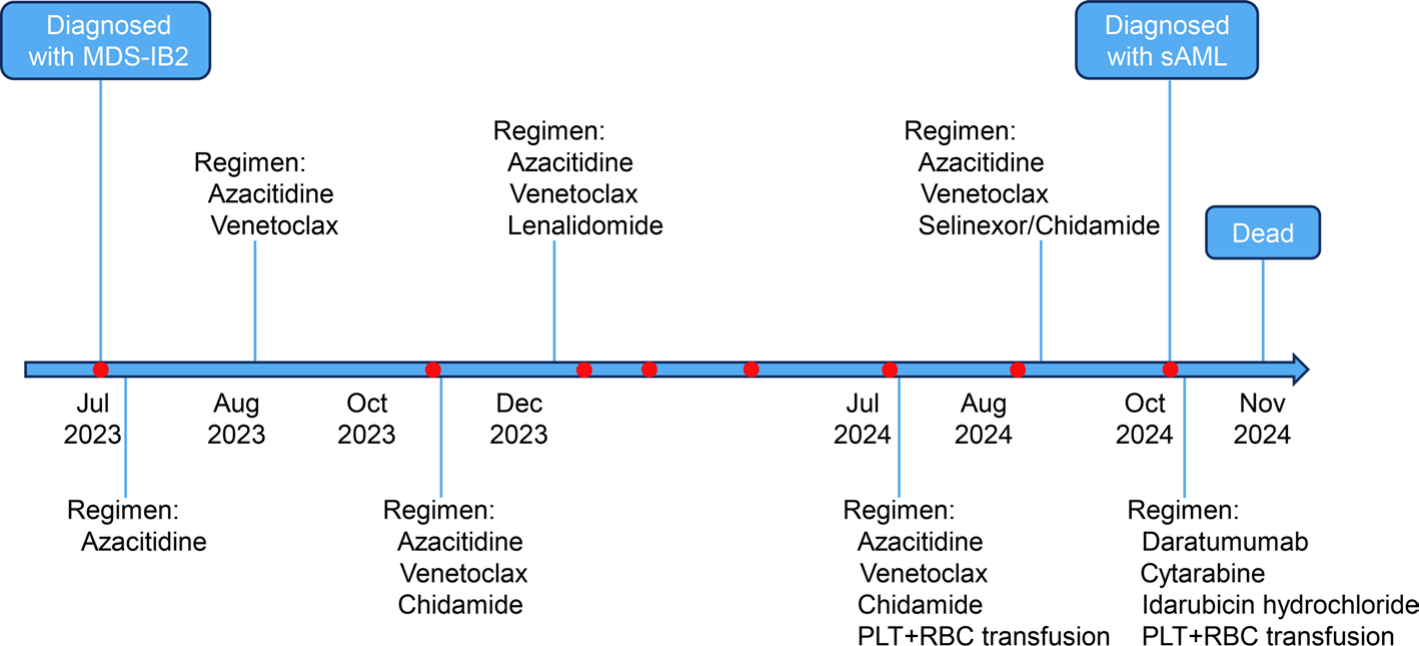
Timeline of diagnosis and treatment of the patient. The red solid dots represent the time points for genetic testing via next-generation sequencing platform. MDS-IB2, myelodysplastic neoplasm with increased blasts 2; sAML, secondary acute myeloid leukemia; PLT, Platelets; RBC, red blood cells

**Fig. 2:**
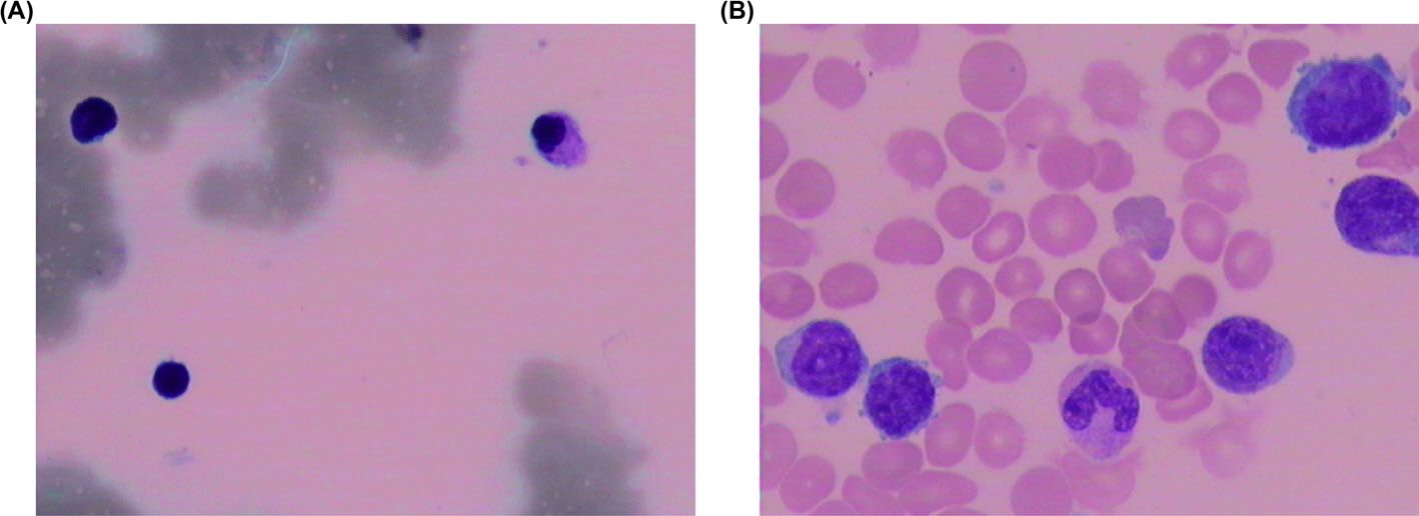
BM examination results showing the morphological feature of MDS (**A**) and sAML (**B**). BM, bone marrow; MDS, myelodysplastic neoplasm; sAML, secondary acute myeloid leukemia

**Fig. 3:**
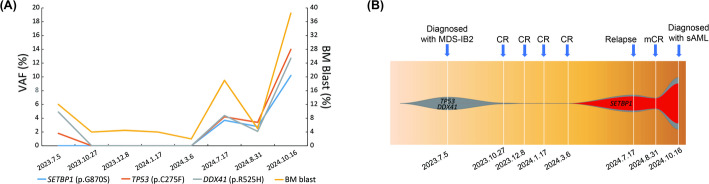
Longitudinal analysis of VAF (**A**) indicating clonal evolution (**B**). The horizontal axis represents follow-up time, and the vertical axis indicates VAF. VAF, variant allele frequency; BM, bone marrow; MDS-IB2, myelodysplastic neoplasm with increased blasts 2; sAML, secondary acute myeloid leukemia; CR, complete remission; mCR, BM CR

A 62-year-old man presented to local hospital with complaints of fatigue, dizziness and palpitations on July 2, 2023 (Fig. 1). A routine examination revealed pancytopenia, and the patient was admitted to our hospital on the same day with the diagnosis of pancytopenia. A CBC showed a white blood cell count (WBC) of 1.8 × 10^9^/L, hemoglobin (Hb) of 94 g/dL, and platelets (PLT) of 34 × 10^9^/L. Subsequent BM examination showed active marrow hyperplasia, and granulocyte proliferation with 12.0% blasts (Fig. 2A). BM immunohistochemical results showed CD34+ (10%). Multi-parameter flow cytometry revealed increased myeloid blasts, and abnormal cells constituted 10.7% of nucleated cells with CD34 (+), CD117 (+), HLA-DR (+), CD64 (−), CD19 (−), CD14 (−), CD7 (−), and CD11b (−). BM chromosomal karyotype analysis indicated a normal male karyotype, 46, XY [20]. He was diagnosed with MDS with increased blasts 2 (MDS-IB2) via the criteria of the 2022 WHO classification of myeloid and histiocytic/dendritic neoplasms [4]. Subsequently, the patient underwent a 521-gene targeted hematologic disorder panel genetic test via next-generation sequencing (NGS) (Table S1), which revealed mutations in *DDX41* (p.R525H, VAF: 4.9%) and *TP53* (p.C275F, VAF: 1.8%, single hit mutation) (Fig. 3). Azacitidine and best supportive care (BSC) were both administered on Jul 6, 2023. Venetoclax was subsequently added on Aug 8, 2023. Chidamide and lenalidomide were additionally administered on Oct 28, 2023 and on Dec 9, 2023, respectively.

Between Sep 18, 2023 and Mar 6, 2024, the responses of this patient were all BM complete remission (mCR) via the IWG 2006 response criteria. During this period, the patient underwent four genetic tests, and mutations in *DDX41* (p.R525H) and *TP53* (p.C275F) were completely eliminated (Fig. 3). On Jul 16, 2024, BM examination showed granulocyte proliferation with 19.0% blasts, indicating patient was currently experiencing disease relapse. NGS uncovered a novel mutated *SETBP1* (p.G870S), and two mutations that were identified at diagnosis. Meanwhile, the treatment plan for the patient was changed to azacitidine, venetoclax, chidamide and BSC. On Aug 29, 2024, BM examination showed granulocyte proliferation with 3.0% blasts, demonstrating that the patient had achieved mCR. The patient’s treatment plan was changed to azacitidine, venetoclax, selinexor and BSC. Due to the worsening vomiting symptoms and the inability to eat of the patient, selinexor was subsequently replaced with chidamide.

On Oct 14, 2024, the BM smear showed 38.5% blasts (Fig. 1C), indicating this patient’s condition had progressed. CBC test revealed WBC of 1.7 × 10^9^/L, Hb of 2.0 g/L, and PTL of 24 × 10^9^/L. Flow cytometry analysis showed that 25.7% of nucleated cells were abnormal with CD34 (+), CD38 (+), CD117 (+), HLA-DR (+), CD64 (−), CD19 (−), CD14 (−), CD7 (−), and CD11b (−). NGS analysis revealed that the VAFs of these three gene mutations, including *SETBP1* (p.G870S), *DDX41* (p.R525H) and *TP53* (p.C275F, single hit mutation), increased to 10.2%, 14.0%, and 12.7%, respectively. He was diagnosed with sAML. The continuous results of genetic test results suggested that the newly emerged *SETBP1* (p.G870S) might be associated with the transformation of MDS into AML. The patient refused to undergo hematopoietic stem cell transplantation due to financial constraints. The patient was subsequently treated with daratumumab, cytarabine, idarubicin hydrochloride and BSC.

On Nov 3, 2024, the patient entered a light coma and the condition further deteriorated. The patient’s family members discussed the situation, decided to refuse treatment and requested discharge. Unfortunately, the patient passed away on the same day.

## Discussion

This case presents a compelling narrative of a 62-year-old male who transitioned from MDS-IB2 to sAML with multiple dynamic genetic mutation detections. The importance of this case lies not only in the patient’s clinical course but also in the insights it provides into the genetic landscape and treatment challenges of MDS and sAML. Understanding this progression illuminates the complexity of managing hematological malignancies, which often evolve from one entity to another. This highlights the significance of vigilant monitoring and tailored treatment strategies.

The existing literature underscores the complexity of MDS and its potential evolution into sAML. It emphasizes that genomic alterations play a critical role in the prognosis and treatment response of these patients [5, 6, 9]. Specifically, mutations in genes such as *DDX41* and *TP53* have been implicated in the development and progression of these diseases. *DDX41* is exemplary of RNA helicase genes affected by somatic mutations, and mutated *DDX41* caused altered pre-mRNA splicing and RNA processing, indicating that *DDX41* constitutes a family of tumor suppressor genes [10–12]. Multiple studies have demonstrated that *TP53* mutations are associated with poor prognosis and resistance to therapies in myeloid hematologic disorders [13–17]. In this case, the presence of the *DDX41* and *TP53* mutations initially suggested a complex course requiring aggressive treatment. The initial treatment showed that the patient achieved CR and completely eliminated the mutant genes. Subsequent identification of a novel *SETBP1* mutation further complicated the clinical picture, and this patient’s disease progressed, underscoring the need for continuous genomic monitoring. Consistent with a previous study [18], the *SETBP1* mutation was acquired only during leukemic evolution, not at initial presentation. Basic research has demonstrated the secondary nature of *SETBP1* mutations through mutational analysis of colonies derived from individual progenitor cells [18]. We found that as the proportion of BM blasts increased, the VAFs of the mutated genes also increased simultaneously, suggesting that VAF reflects the patient’s tumor burden. Unlike previous *SETBP1* associated MDS/AML studies, we found that this patient’s mutations from relapse and transformation into sAML included both *DDX41* and *TP53* mutations, other than previously reported mutated *ASXL1*, *SRSF2*, *U2AF1*, *RUNX1*, etc [18–20]. Previous literature has not reported the prognostic value of the combined mutation of these three genes. The joint effect may have contributed to the rapid progression of the disease. The patient underwent eight genetic mutation tests from the time of being diagnosed with MDS until the final diagnosis of sAML, indicating that continuous monitoring of genetic mutations reflects changes in the patient’s therapeutic response.

The patient’s transition from MDS to sAML highlights the challenges faced in therapeutic management. The patient’s treatment involved included azacitidine and venetoclax, both showing promise in managing MDS. However, as the disease progressed, new mutations emerged, and the patient’s clinical condition deteriorated, necessitating a reevaluation of his treatment plan. This reflects a broader challenge: patients with sAML often experience limited responses to conventional therapies. The decision to modify the treatment plan according to the evolving genetic landscape exemplifies a key aspect of personalized medicine in hematological malignancies. Ultimately, as the patient’s condition worsened and treatment responses became increasingly limited, the family decided to refuse further aggressive treatment. This situation highlights the importance of integrating palliative care principles into managing patients with hematological malignancies to ensure treatment decisions align with patients’ goals and values. In the future, in order to better study the process of cloning evolution, multi-omics research needs to be conducted [21].

## Conclusion

In summary, this case firstly illustrates the combined mutation of *SETBP1*, *DDX41* and *TP53* caused the rapid progression of the disease. The findings emphasize the necessity of dynamic monitoring of genetic mutations, which allows us to better inform clinical practice and enhance care for patients facing hematological malignancies.

## Supplementary Information

Supplementary Material 1.

## Data Availability

The datasets used and/or analysed during the current study are available from the corresponding author on reasonable request.
