# Supplementary material for: Case report: Dynamic genetic profiles reveal a patient with myelodysplastic neoplasm transforming into acute myeloid leukemia
Source: Discov Oncol. 2026 Mar 27;17:701. doi: 10.1007/s12672-026-04863-y (PMC13144453; doi:10.1007/s12672-026-04863-y)
Supplement: Supplementary file 1 — Supplementary Material 1. [file 12672_2026_4863_MOESM1_ESM.docx]

**Table S1. The 521-gene sequencing panel**

| *ABL1* | *FANCG* | *PIGA* | *TRAF3* | *CTCF* | *HIST1H2BK* | *NUDT15* | *SOCS1* |
| --- | --- | --- | --- | --- | --- | --- | --- |
| *ACD* | *FANCI* | *PIK3CD* | *TSR2* | *CTLA4* | *HIST1H3B* | *OR10A2* | *SOD2* |
| *ANKRD26* | *FANCL* | *PIM1* | *U2AF1* | *CTNNB1* | *HIST1H3D* | *P2RY8* | *SOS1* |
| *ARC* | *FANCM* | *PLCG1* | *UBE2T* | *CUX1* | *HIST1H3H* | *PBRM1* | *SPEN* |
| *ARID1A* | *FAS* | *PLCG2* | *VAV1* | *CXCR5* | *HIST1H3J* | *PCBP1* | *SPI1* |
| *ARID1B* | *FBXW7* | *POT1* | *VHL* | *CYBA* | *HIST1H4E* | *PDCD1* | *SRCAP* |
| *ARID2* | *FGFR3* | *PPM1D* | *WAS* | *CYP2B6* | *HIST1H4J* | *PDE4DIP* | *SRP54* |
| *ASXL1* | *FLT3* | *PRDM1* | *WRAP53* | *CYP2C19* | *HIST2H2BE* | *PDGFRA* | *SRSF1* |
| *ASXL2* | *FOXO1* | *PRKCB* | *WT1* | *CYP2C8* | *HLA-DRB1* | *PDS5B* | *STAG1* |
| *ASXL3* | *FYN* | *PRPF8* | *XRCC2* | *CYP3A4* | *HNRNPU* | *PIK3CA* | *STAT5A* |
| *ATM* | *G6PC3* | *PTEN* | *ZBTB7A* | *CYP3A5* | *HVCN1* | *PIK3R1* | *STAT6* |
| *B2M* | *GATA1* | *PTPN1* | *ZEB2* | *DARS* | *IFNL3* | *PLXNB3* | *STIM1* |
| *BCL2* | *GATA2* | *PTPN11* | *ZRSR2* | *DCAF6* | *IGLL5* | *PML* | *STX11* |
| *BCL6* | *GATA3* | *RAD21* | *ABCA13* | *DCDC1* | *IL13RA1* | *PMS2* | *STXBP2* |
| *BCOR* | *GFI1* | *RAD51* | *ABCB1* | *DCTD* | *IL1RAPL2* | *PNPLA3* | *SUZ12* |
| *BCORL1* | *GNA13* | *RAD51C* | *ABCC2* | *DDX10* | *IL4R* | *POSTN* | *TAF1* |
| *BIRC3* | *GNAI2* | *RAF1* | *ABCG2* | *DDX11* | *IL6* | *POU2AF1* | *TBL1XR1* |
| *BLM* | *HAX1* | *RB1* | *ACTB* | *DHX30* | *IMPDH2* | *POU2F2* | *TCL1A* |
| *BLNK* | *HNRNPA2B1* | *RHOA* | *ACTG1* | *DHX58* | *IRF2BP2* | *PPFIA2* | *TCTN2* |
| *BRAF* | *ID3* | *RPL11* | *ADD2* | *DIS3* | *IRF4* | *PRF1* | *TFAP4* |
| *BRCA1* | *IDH1* | *RPL15* | *ADGRV1* | *DNM2* | *IRF8* | *PROX1-AS1* | *TLR2* |
| *BRCA2* | *IDH2* | *RPL23* | *ADSL* | *DNMT1* | *IRX2* | *PRPS1* | *TMEM30A* |
| *BRIP1* | *IKZF1* | *RPL26* | *AKAP6* | *DOK5* | *ITPA* | *PRUNE2* | *TMSB4X* |
| *BTK* | *IKZF2* | *RPL27* | *AKT3* | *DROSHA* | *ITPR3* | *PTPN6* | *TNF* |
| *CALR* | *IKZF3* | *RPL31* | *ALK* | *DTX1* | *JARID2* | *PTPRC* | *TNFSF14* |
| *CARD11* | *IL7R* | *RPL35A* | *AOC2* | *DYNC2H1* | *KDM5C* | *PTPRD* | *TOX* |
| *CBL* | *INO80* | *RPL5* | *ARID3A* | *EBF1* | *KIAA1671* | *PTPRM* | *TPMT* |
| *CCND1* | *INPP5D* | *RPS10* | *ATP6AP1* | *ECT2L* | *KLHL14* | *PTPRN2* | *TRAF2* |
| *CCR4* | *ITPKB* | *RPS17* | *ATP6V1B2* | *EED* | *KLHL6* | *RAB27A* | *TRRAP* |
| *CCR7* | *JAK1* | *RPS19* | *ATRX* | *EEF1A1* | *KMT2B* | *RARA* | *TSPAN19* |
| *CD28* | *JAK2* | *RPS24* | *BACH2* | *EGR1* | *LEF1* | *RBBP6* | *TTN* |
| *CD58* | *JAK3* | *RPS26* | *BCL11A* | *EGR2* | *LINC00251* | *REL* | *TYK2* |
| *CD79A* | *JUNB* | *RPS27* | *BCL11B* | *EIF4A1* | *LRRN3* | *RELN* | *U2AF2* |
| *CD79B* | *KANSL1* | *RPS28* | *BCL7A* | *ERBB3* | *LTB* | *RFX7* | *UBE2A* |
| *CDKN1B* | *KDM6A* | *RPS29* | *BCR* | *ERBB4* | *LUC7L2* | *RGS1* | *UGT1A1* |
| *CDKN2A* | *KIT* | *RPS7* | *BIRC6* | *ERCC1* | *LYN* | *RIT1* | *UGT1A8* |
| *CDKN2B* | *KLF2* | *RRAGC* | *BMP7* | *ERG* | *LYST* | *ROBO1* | *UNC13D* |
| *CEBPA* | *KMT2A* | *RTEL1* | *BRCC3* | *FAM46C* | *MAGEC2* | *ROBO2* | *USB1* |
| *CHD8* | *KMT2C* | *RUNX1* | *BRINP3* | *FAT1* | *MAGT1* | *RP1L1* | *USH2A* |
| *CREBBP* | *KMT2D* | *SAMD9* | *BTG1* | *FAT4* | *MAP3K14* | *RPL10* | *USP7* |
| *CSF1R* | *KRAS* | *SAMD9L* | *BTG2* | *FBXO11* | *MED12* | *RPL18* | *VMA21* |
| *CSF3R* | *MAP2K1* | *SBDS* | *CARMIL2* | *FCGR3A* | *MGA* | *RPS15* | *VMP1* |
| *CTC1* | *MAPK1* | *SETBP1* | *CCL4* | *FGA* | *MME* | *RRM1* | *WDFY3* |
| *CXCR4* | *MBD4* | *SF3B1* | *CCND2* | *FGB* | *MPEG1* | *RRM2* | *XIAP* |
| *DDX3X* | *MECOM* | *SGK1* | *CCND3* | *FGFR1* | *MS4A1* | *RRM2B* | *XPO1* |
| *DDX41* | *MEF2B* | *SH2B3* | *CD274* | *FGG* | *MSH2* | *S1PR2* | *XRCC5* |
| *DHX15* | *MET* | *SH2D1A* | *CD70* | *FPGT* | *MTHFR* | *SAMHD1* | *YLPM1* |
| *DKC1* | *MFHAS1* | *SLX4* | *CD83* | *FZD3* | *MTOR* | *SCG3* | *YTHDF2* |
| *DNAJC21* | *MLH1* | *SMARCA4* | *CDA* | *GCSAM* | *MTRR* | *SERPINB3* | *YY1* |
| *DNMT3A* | *MPL* | *SMC1A* | *CDC25C* | *GDF11* | *MYB* | *SERPINE1* | *ZC3H12A* |
| *DUSP2* | *MSH6* | *SMC3* | *CDKN2C* | *GNAS* | *MYC* | *SETD1A* | *ZCCHC11* |
| *EFL1* | *MYBBP1A* | *SRP72* | *CEP72* | *GNB1* | *NCOR1* | *SETD1B* | *ZFP36L1* |
| *ELANE* | *MYD88* | *SRSF2* | *CHD2* | *GPR37* | *NCOR2* | *SETD2* | *ZFP36L2* |
| *EP300* | *NAF1* | *STAG2* | *CIITA* | *GRB2* | *NFATC2* | *SETD5* | *ZMYM3* |
| *EPHA7* | *NF1* | *STAT3* | *CISH* | *GRM7* | *NFKBIA* | *SETDB2* | *ZNF217* |
| *EPOR* | *NHP2* | *STAT5B* | *CLGN* | *GSTM1* | *NFKBIE* | *SF1* | *ZNF292* |
| *ERCC4* | *NOP10* | *TCF3* | *CNKSR2* | *GSTP1* | *NLRC4* | *SGPP1* | *ZNF423* |
| *ETNK1* | *NOTCH1* | *TERC* | *CNOT3* | *GTF2I* | *NLRP8* | *SIN3A* | *ZNF608* |
| *ETV6* | *NOTCH2* | *TERT* | *COL6A3* | *HDAC9* | *NOL9* | *SLC22A1* | *ZNF80* |
| *EZH2* | *NPM1* | *TET2* | *COQ7* | *HIST1H1B* | *NOTCH3* | *SLC29A1* |  |
| *FANCA* | *NRAS* | *TET3* | *CPA2* | *HIST1H1C* | *NOTCH4* | *SLCO1A2* |  |
| *FANCB* | *PALB2* | *TINF2* | *CRBN* | *HIST1H1D* | *NR3C1* | *SLCO1B1* |  |
| *FANCC* | *PARN* | *TNFAIP3* | *CRIP1* | *HIST1H1E* | *NRXN3* | *SLITRK3* |  |
| *FANCD2* | *PAX5* | *TNFRSF14* | *CRLF2* | *HIST1H2AC* | *NSD2* | *SMAD4* |  |
| *FANCE* | *PDGFRB* | *TNFRSF1B* | *CSF2RB* | *HIST1H2AM* | *NT5C2* | *SMARCB1* |  |
| *FANCF* | *PHF6* | *TP53* | *CSNK1A1* | *HIST1H2BC* | *NTRK1* | *SNTB2* |  |
